# Clinical Relevance of Serum Kyn/Trp Ratio and Basal and IFNγ-Upregulated IDO1 Expression in Peripheral Monocytes in Early Stage Melanoma

**DOI:** 10.3389/fimmu.2021.736498

**Published:** 2021-09-07

**Authors:** Annabel Meireson, Liesbeth Ferdinande, Marc Haspeslagh, Benjamin Hennart, Delphine Allorge, Piet Ost, Nora Sundahl, Mathieu Spaas, Annelies Demeyer, Lieve Brochez

**Affiliations:** ^1^Cancer Research Institute Ghent (CRIG), Ghent University, Ghent, Belgium; ^2^Dermatology Research Unit, Ghent University Hospital, Ghent, Belgium; ^3^Department of Pathology, Ghent University Hospital, Ghent, Belgium; ^4^Dermpat, Ghent, Belgium; ^5^Le Centre Hospitalier Universitaire de Lille (CHU), Unité Fonctionnelle de Toxicologie, Lille, France; ^6^Université de Lille, ULR 4483 – IMPECS – IMPact de l’Environnement Chimique sur la Santé humaine, Lille, France; ^7^Department of Radiation Oncology and Experimental Cancer Research, Ghent University Hospital, Ghent, Belgium

**Keywords:** early stage melanoma, biomarker, IDO1, Kyn/Trp, tryptophan metabolism, monocytes, immune cells, liquid biopsy

## Abstract

Immune escape is an early phenomenon in cancer development/progression. Indoleamine 2,3-dioxygenase 1 (IDO1) is a normal endogenous mechanism of acquired peripheral immune tolerance and may therefore be tumor-promoting. This study investigated the clinical relevance of IDO1 expression by immune cells in the lymph nodes and blood and of the serum kynurenine/tryptophan (Kyn/Trp) ratio in 65 systemic treatment naïve stage I-III melanoma patients. Blood samples were collected within the first year of diagnosis. Patients had a median follow-up of 61 months. High basal IDO1 expression in peripheral monocytes and low IFNγ-induced IDO1 upregulation correlated with worse outcome independent from disease stage. Interestingly studied factors were not interrelated. During follow-up, the risk of relapse was 9% (2/22) in the subgroup with high IFNγ-induced IDO1 upregulation in monocytes. In contrast, if IDO1 upregulation was low, relapse occurred in 30% (3/10) of patients with low basal IDO1 expression in monocytes and in 61.5% (8/13) in the subgroup with high basal IDO1 expression in monocytes (Log-Rank test, p=0.008). This study reveals some immune features in the blood of early stage melanoma that may be of relevance for disease outcome. These may offer a target for sub-stratification and early intervention.

## 1 Introduction

The incidence of cutaneous melanoma increases worldwide and is responsible for 75% of skin cancer-related deaths (1). New insights in immuno-oncology and the subsequently developed immunotherapies have caused a major breakthrough in the oncology field in general and more specifically in the management of metastatic melanoma in the last decade, creating the hope of curing (metastatic) cancer.

Biomarker studies in immuno-oncology have mainly addressed stage IV melanoma patients receiving immunotherapy. However, a significant proportion of melanoma-caused deaths originally present with early stage disease (2–4). Except for assessment of tumor-infiltrating lymphocytes (TILs) in tumor tissue, which has been recognized to provide prognostic information in several cancer types (5–7), little attention has been focused on immune dysfunctions in early stage cancer. Importantly, the adaptive immune response has been shown to be strongest in pre-invasive stages compared to the metastasized stage of carcinogenesis (8–11). The interaction between the tumor and the host immune system is acknowledged as one of the hallmarks of cancer development and progression (12). Studies in early stage cancer demonstrated that tumors harness various mechanisms to escape immune surveillance during their evolution (13, 14). Immune evasion through upregulation of immune checkpoints such as indoleamine 2,3-dioxygenase (IDO1), cytotoxic T-lymphocyte-associated protein 4 (CTLA-4) and programmed death-ligand 1 (PD-L1) has been identified as an evolutionary trajectory during pre-invasive stages of lung cancer (15).

IDO1 is an intracellular enzyme that initiates the rate-limiting step in the catabolism of the essential amino acid tryptophan (Trp) to kynurenine (Kyn). As the promoter region of *IDO1* consists of several IFN-stimulated response elements (ISREs) and gamma activation sequences (GAS), interferon gamma (IFNγ) is well-recognized as the predominant inducer of IDO1 upregulation (16, 17). Increased conversion of Trp to Kyn has been demonstrated to inhibit T-cell proliferation and activation (18–20). IDO1 can be expressed by a variety of cells in the broad tumor micro-environment e.g. tumor, endothelial and stromal cells (21). A negative prognostic value of tumoral IDO1 expression has been reported in a broad spectrum of malignancies (22) and some studies correlated tumoral IDO1 expression with serum Kyn/Trp (23, 24). However, the role of IDO1 expression in immune cells and its relation to the serum Kyn/Trp ratio has not been investigated until now.

In this study the impact of the serum Kyn/Trp ratio and IDO1 expression by immune cells of the lymph node and peripheral blood compartment on clinical outcome was investigated in a cohort of stage I-III melanoma patients with long-term follow-up. In addition, peripheral blood mononuclear cells (PBMCs) were cultivated *in vitro* with IFNγ in order to compare IDO1 upregulation in patients with stable *versus* progressive disease.

## 2 Materials and Methods

### 2.1 Patient Cohort

Sixty-five drug-therapy naïve melanoma patients with disease stage I (n=18), stage II (n=21) or stage III (n=26) were included. This study was approved by the Ghent University Hospital Ethics Committee. All patients gave written informed consent before inclusion in accordance with the Helsinki Declaration.

### 2.2 Sample Collection

Blood samples were collected at consultation during follow-up of primary melanoma. Serum (n=65) was isolated and cryopreserved at -80°C after centrifugation for 10 min at 1500 rpm. Peripheral blood mononuclear cells (PBMCs, n=47) were obtained from ethylenediaminetetraacetic acid (EDTA) anticoagulated whole blood by Ficoll density gradient centrifugation for 20min at 2200 rpm and cryopreserved at -196°C for batch analysis.

For 41 patients (stage I: n=9, stage II: n=16, stage III: n=16) who underwent a sentinel lymph node procedure at the Ghent University Hospital, formalin-fixed paraffin-embedded (FFPE) material of this resection was available for immunohistochemistry.

### 2.3 Determination of Serum Trp and Metabolites

Trp, its catabolite Kyn and other downstream metabolites were quantified according to previously published methods (25, 26), with slight modifications. Serum samples (50 µL) were extracted using 50 µL acetonitrile containing Trp-D5 (50µM, CDN Isotopes, Pointe-Claire, QC, Canada) as an internal standard. The samples were centrifuged (8 min, 11.800 rpm, 4°C) and the supernatants (50 µL) were added to deionized water (600 µL). 15 µL of this mixture was injected onto an ultra-performance liquid chromatography coupled to tandem mass spectrometry (UPLC-MS/MS) system (Acquity TQ-S Detector, Waters, Milford, MA) equipped with a HSS C18 column. Ions of each analyzed compound were detected in a positive ion mode using multiple reaction monitoring.

Kyn/Trp values from melanoma stage I-III were compared to those of 20 stage IV melanoma patients before start of a phase 2 trial testing nivolumab combined with stereotactic body radiotherapy (NCT02821182) (27) and of 94 healthy subjects of a previously published cohort (28).

### 2.4 Immunohistochemistry

#### 2.4.1 IDO1 Staining

Four µm sections were cut from FFPE tissue blocks and were heated for 30 min at 60°C. IDO1 staining was executed on an automated and validated Leica Bond-Max stainer (Leica Microsystems, Wetzlar, Germany). In brief, after dewaxing heat-induced epitope retrieval was performed in a high pH EDTA buffer (Bond Epitope Retrieval 2, Leica Microsystems) for 30 min. Slides were incubated with the primary IDO antibody (clone 10.1, Merck, Darmstadt, Germany) at a 1:50 dilution for 15 min and the antibody was detected *via* BOND Polymer Refine Red Detection kit (Leica Microsystems). Slides were then counterstained with hematoxylin.

IDO1 expression was scored in immune cells in the paracortex and in the sinuses of the lymph node, excluding the germinal centers. In the paracortex, the intensity of IDO1 staining was scored semiquantitatively using a four-tiered grading system (**Figure 2A**): no expression (0), weak expression (1+), moderate expression (2+) or strong expression (3+). For further analysis, IDO1 expression in the paracortex was dichotomized into an IDO1-low group (0 and 1+) and an IDO1-high group (2+ and 3+). In the sinuses, IDO1 staining was scored as ‘low’ or ‘high’ (**Figure 2B**).

#### 2.4.2 PD-L1 Staining

Four µm sections were cut from FFPE tissue blocks. After deparaffinization, antigen retrieval was performed by CC1 antigen retrieval solution (pH 8.0, Ventana Medical systems, Tuczon, AZ, USA) on the Ventana BenchMark Ultra automated slide stainer. Slides were incubated with the primary PD-L1 antibody (anti-PD-L1, clone 22C3, Merck) at a dilution of 1/100 for 32 min, followed by visualization with the OptiView DAB IHC Kit and OptiView Amplification Kit (Ventana Medical systems). The specimens were then counterstained with haematoxylin and bluing reagent (Ventana Medical systems) and coverslipped.

PD-L1 expression was scored in immune cells in the paracortex and in the sinuses of the lymph node, excluding the germinal centers. Similar to IDO1 scoring, the intensity of PD-L1 staining in the paracortex was evaluated according to a four-tiered grading system (**Supplementary Figure 3A**): no expression (0), weak expression (1+), moderate expression (2+) or strong expression (3+). PD-L1 expression in the paracortex was dichotomized into an PD-L1-low group (0 and 1+) and an PD-L1-high group (2+ and 3+). In the sinuses, PD-L1 staining was scored as ‘low’ or ‘high’ (**Supplementary Figure 3B**).

### 2.5 Flow Cytometry

Cryopreserved PBMCs (n=45) were thawed and washed in RPMI 1640 medium supplemented with Glutamax (2.05 mM), 10% FCS and penicillin (100U/mL) – streptomycin (100µg/mL). Per patient, 2 x 10^6^ cells were cultured in a 24-well plate in medium with and without IFNγ (R&D systems, Minneapolis, MN, USA) at 50 ng/mL for 16 hours at 37°C. Cells were then collected and washed in PBS prior to incubation with human Fc blocker (Miltenyi, Madrid, Spain), eFluor506 live/dead marker (eBioscience, San Diego, CA, USA) and antibodies for surface staining for 30 min at 4°C. The following antibodies were used: anti-CD14-Pacific Blue (clone 63D3, Biolegend, San Diego, CA, USA), anti-CD16-BV605 (clone 3G8, BD Biosciences, San Jose, CA, USA), anti-HLA-DR-AF700 (clone G46-6, BD Biosciences), anti-PD-L1-PE/Cy7 (clone 29E.2A3, Biolegend). Cells were fixed and permeabilized with Foxp3 Transcription Factor Staining Buffer Set (eBioscience) in accordance with the manufacturer’s protocol and subsequently stained intracellularly with anti-IDO-PE (clone #700838, R&D systems) for 30 min at room temperature. Labeled cell suspensions were acquired on a BD Fortessa X20 (BD Biosciences) and data was analyzed with FlowJo 10.6.2 software. Gating strategies are depicted in **Supplementary Figure 1**.

### 2.6 Cytokine Detection in Supernatants

Thawed PBMCs (n=47) were seeded in a 96-well plate (0.5 x 10^6^ cells/well) in RPMI 1640 medium (supplemented with Glutamax, FCS and penicillin – streptomycin) and stimulated with anti-CD3/CD28 (Dyna beads Human T-activator, ThermoFisher Scientific, Waltham, MA, USA) for 16 hours at 37°C. Cell-free supernatants was collected and stored at -80°C until cytokine measurement. Magnetic multiplex immunoassay (Luminex Technology, R&D systems) was performed on supernatants according to manufacturer’s instructions using a customized panel, including CD137, CD40 Ligand, Fas Ligand, Granzyme A, Granzyme B, IFNγ, IL-1β, IL-2, IL-4, IL-6, IL-10, IL-17, IL-21, PD-L1 and TNFα. Concentrations were measured on a Bio-Plex 200 Array Reader (Bio-Rad, Hercules, CA, USA). Concentrations were divided by the percentage of viable lymphocytes in order to normalize for variations in lymphocyte frequency between patients.

### 2.7 Statistics

A chi square test was employed to test for association between two categorical variables. Correlations between continuous variables were determined by Spearman’s correlation coefficient. A continuous variable was compared between two groups by a two-tailed Mann–Whitney *U*-test. Progression free survival (PFS) was defined as the time from inclusion to disease progression or death from any cause. PFS curves were estimated using the Kaplan-Meier method by dichotomizing variables through their median value. Survival curves between patients with high (above the median) and low (below the median) frequencies of the variable were compared using a Log-Rank test. Cox regression models were used to perform univariate and multivariate analysis. To compare immunologic variations between unstimulated and stimulated conditions, a Wilcoxon matched-pairs signed-ranks test was executed. The effect of IFNγ-stimulation on IDO1 expression was represented by the median fluorescence intensity (MFI) of the stimulated condition minus the MFI of the unstimulated condition. Statistical analyses were performed using IBM SPSS v27 and all tests were performed two-sided; *p* < 0.05 was considered to be statistically significant. Graphs were plotted with Graphpad Prism (GraphPad software Inc., San Diego, CA, USA).

## 3 Results

### 3.1 Serum Kyn/Trp Ratio and Relation With PFS

Sixty-five drug-therapy naive melanoma patients (18 stage I, 21 stage II and 26 stage III) with a median follow-up time of 61.0 months after diagnosis (IQR: 37.5-80.0) were included in this study (**Table 1**). Nineteen patients (29.2%) demonstrated progressive disease during follow-up time, which occurred with a median time of 21.0 months (IQR: 9.0-66.0) after diagnosis.

**Table 1:** Patient characteristics.

Characteristics		N = 65
Age at inclusion (years)		
	Median (IQR)	53.00 (40.5 - 64.5)
**Sex**		
	Female/Male	27/38
**AJCC disease stage at time of inclusion (progression/death)**		
	IA	2 (-/-)
	IB	16 (1/1)
	IIA	8 (2/2)
	IIB	10 (5/1)
	IIC	3 (1/1)
	IIIA	10 (1/-)
	IIIB	13 (6/2)
	IIIC	3 (3/2)
**Breslow thickness (mm)**		
	Median (IQR)	2.10 (1.21 - 3.90)
**Ulceration**		
	No/Yes	45/16
	Missing	4
**Follow-up time after inclusion (months)**		
	Median (IQR)	56.0 (33.5 - 71.0)

**Figure 1:**
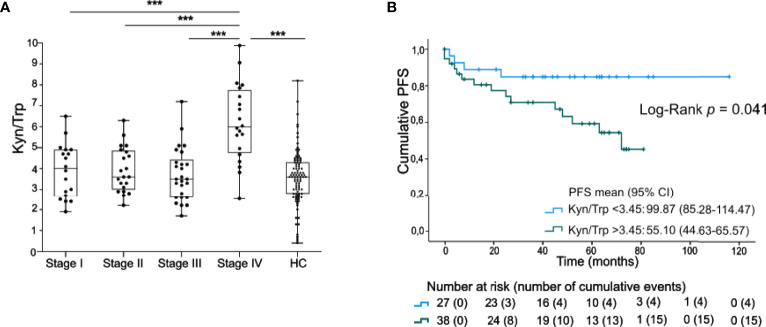
Serum Kyn/Trp ratio is negatively prognostic in early staged melanoma. **(A)** Serum ratio of Kyn to Trp (presented values are Kyn/Trp x 100) in stage I-IV melanoma patients and healthy controls (HC). *P* value calculated using two-sided Mann-Whitney U test. ***p < 0.001. Whiskers of boxplots extend to the minimum and maximum data point, with the horizontal line indicating the median. **(B)** Kaplan-Maier estimate of PFS stratified according to low serum Kyn/Trp ratio (<3.45) or high serum Kyn/Trp ratio (>3.45). *P* value calculated using Log-Rank test.

Trp and its metabolites were profiled in serum samples. No significant differences for serum Trp nor for the Kyn/Trp ratio as compared to healthy controls (n=94) were noted (**Figure 1A**, **Supplementary Figure 2**). Kyn concentrations and Kyn/Trp were however significantly higher in a group of 20 stage IV patients as compared to the group of stage I-III patients (**Supplementary Figure 2**, **Figure 1A**). In stage I-III, the Kyn levels and the Kyn/Trp ratio were significantly higher in males compares to females (resp. p<0.001 and p=0.009) whereas only a trend to higher Trp concentrations in males was noticed (p=0.061). In addition, the Kyn/Trp ratio was positively correlated with age of the patient (Spearman’s CC: 0.381, p=0.002). The levels of Trp and Kyn were not, or only borderline, correlated with age (resp. Spearman’s CC:-0.143, p=0.257 and CC:0.215, p=0.085).

The clinical significance of the serum Kyn/Trp ratio was further examined in 65 patients with stage I-III melanoma. Patients with a low Kyn/Trp ratio (below median) had a significantly higher PFS [99.9 months (95% CI 85.3-114.5)] compared to the subgroup with a high (above median) Kyn/Trp ratio (55.1 months (95% CI 44.6-65.6), Log-Rank test, p=0.041, **Figure 1B**). Of note, progression occurred up to 72 months after blood sample collection [median time to progression after inclusion was 12.0 months (IQR: 4.0-45.0)]. Kyn/Trp remained an independent negative prognostic marker for PFS after adjusting for Breslow thickness but not after adjusting for stage (**Supplementary Table 1**).

### 3.2 IDO1 Expression in Lymph Nodes and Relation to Systemic Immunity

**Figure 2:**
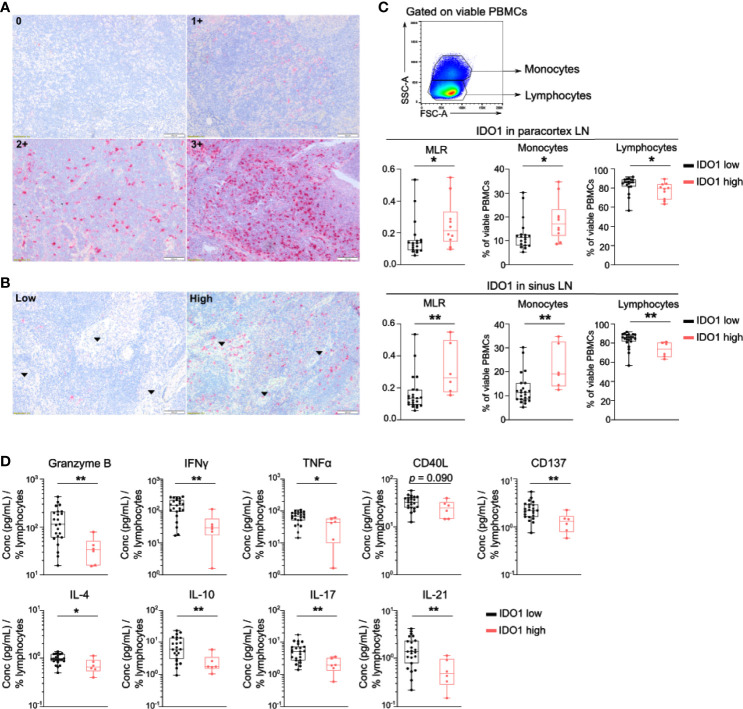
IDO1 is expressed by immune cells in the lymph node and correlates with altered frequencies of circulating monocytes and lymphocytes. **(A)** Representative immunohistochemical images of IDO1 expression in the paracortex and **(B)** in the sinuses (indicated by arrows) of the lymph node. **(C)** (top) Gating strategy of circulating monocytes and lymphocytes in viable PBMCs. (middle) Boxplots with monocyte-to-lymphocyte ratio (MLR), frequency of monocytes and lymphocytes according to IDO1 expression in the paracortex and (bottom) IDO1 expression in the sinuses. **(D)** Boxplots with the concentration of indicated cytokine in supernatants of anti-CD3/CD28 activated PBMCs according to IDO1 expression in the sinuses. The cytokine concentration was divided by the percentage of viable circulating lymphocytes per patient. **(C, D)** *P* value calculated using two-sided Mann-Whitney U test. *p < 0.05, **p < 0.01. Whiskers of boxplots extend to the minimum and maximum data point, with the horizontal line indicating the median.

Next, IDO1 expression by immune cells in lymph node biopsies was evaluated by IHC. IDO1 expression was prominent in immune cells in the paracortex and the sinuses of the lymph node (**Figures 2A, B**). High IDO1 expression in the paracortex (score 2+ and 3+) was present in 41.5% of patients and in the sinuses in 25.0% of patients. High IDO1 expression in the paracortex and especially in the sinuses was associated with a higher monocyte/lymphocyte ratio (MLR) in the peripheral blood (**Figure 2C**). Analysis of cytokine secretion in supernatants of PBMCs activated with anti-CD3/CD28 beads revealed lower levels of IFNγ, Granzyme B, CD137, IL17, IL-21, IL-4, IL10 and TNFα in patients with high IDO1 expression in the sinuses indicating a less pro-inflammatory immune response (**Figure 2D**). Nevertheless, no association of IDO1 expression in the sinuses/the paracortex of the lymph node with PFS was observed (Log-Rank test resp. p=0.985 and p=0.175).

PD-L1 expression by immune cells was also evaluated in these lymph nodes (**Supplementary Figure 3**). Immune cells expressing PD-L1 were detected in the paracortex (score 2+ or 3+ in 22% of patients) and in the sinuses (high expression in 30% of patients). PD-L1 expression was not associated with IDO1 expression in the lymph nodes and no correlation with PFS was observed.

### 3.3 IDO1 Expression in Circulating Immune Cells

**Figure 3:**
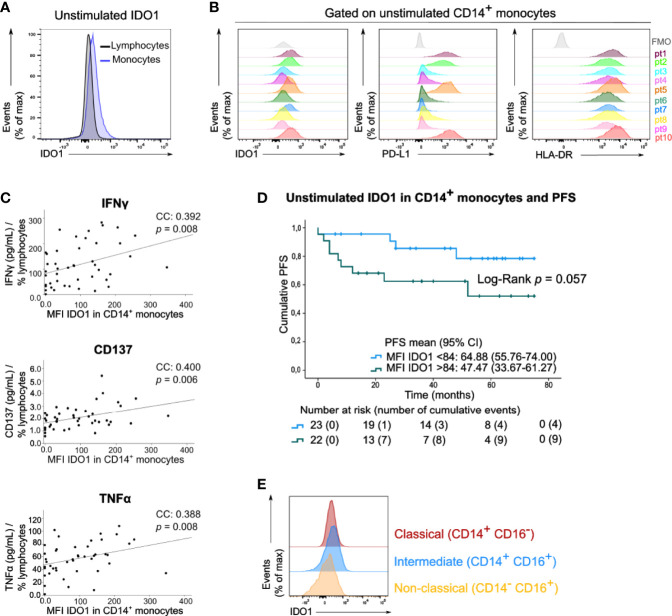
Circulating monocytes express IDO1 under basal conditions. **(A)** Histogram (concatenation of viable cells of 10 samples) with IDO1 expression in circulating lymphocytes and monocytes. **(B)** Histograms with expression of IDO1, PD-L1 and HLA-DR in CD14^+^ monocytes of 10 patients. FMO is depicted in grey. **(C)** Spearman correlation of MFI of IDO1 in CD14^+^ monocytes (FMO was taken into account) and the concentration of IFNγ, CD137 and TNFα in the supernatants of anti-CD3/CD28 activated PBMCs. The cytokine concentration was divided by the percentage of viable circulating lymphocytes per patient. **(D)** Kaplan-Meier estimate of PFS stratified according to low MFI of basal IDO1 (<84) or high MFI of basal IDO1 (>84). *P* value calculated using Log-Rank test. FMO was taken into account for calculation of patients’ MFI of IDO1. **(E)** Histograms (concatenation of viable monocytes of 10 samples) with IDO1 expression in classical, intermediate and non-classical monocytes.

We next examined IDO1 expression in circulating immune cells of the peripheral blood samples of these patients by means of median fluorescent intensity (MFI). In basal (unstimulated) conditions, IDO1 was scarcely expressed by lymphocytes and monocytes (**Figure 3A**). A variable expression of IDO1 in CD14^+^ monocytes was observed across patients. This IDO1 expression was correlated with the expression of PD-L1 and HLA-DR in CD14^+^ monocytes (**Figure 3B**, resp. Spearman’s CC: 0.740, p<0.001 and CC: 0.562, p<0.001). IDO1 expression in CD14^+^ monocytes was positively correlated with IFNγ, CD137 and TNFα levels in supernatants of PBMCs activated with anti-CD3/CD28 beads (**Figure 3C**). Of note, patients with high IDO1 expression in CD14^+^ monocytes tended to have a lower PFS (Log-Rank test, p=0.057, **Figure 3D**). In multivariate analysis, basal IDO1 expression in CD14^+^ monocytes was a negative prognostic marker independent from Breslow thickness and disease stage (**Supplementary Table 1**). Monocytes were classified into three subsets based on CD14 and CD16 expression (**Supplementary Figure 1**). In unstimulated conditions, IDO1 expression was higher in classical (CD14^+^ CD16^-^) and intermediate (CD14^+^ CD16^+^) compared to non-classical (CD14^-^ CD16^+^) monocytes (**Figure 3E**).

**Figure 4:**
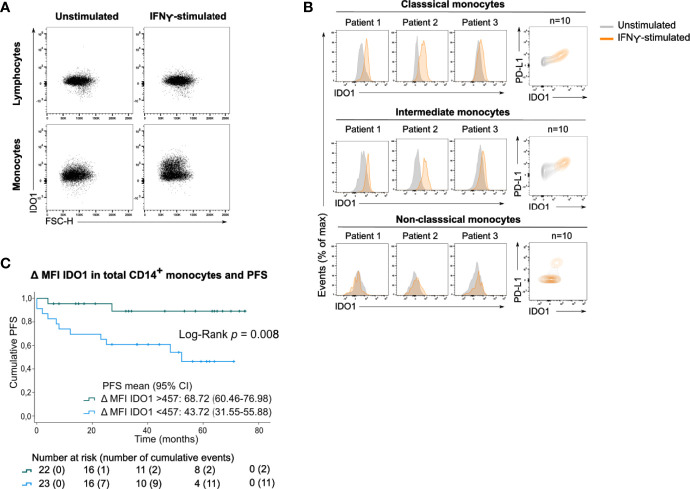
Circulating monocytes upregulate IDO1 upon IFNγ stimulation. **(A)** Dot plots with IDO1 expression in lymphocytes and monocytes without or with IFNγ stimulation (concatenation of viable lymphocytes or viable monocytes of 10 unstimulated or stimulated samples). **(B)** (left) Histograms showing IFNγ-induced IDO1 upregulation in classical, intermediate and non-classical monocytes in 3 patients. (right) Contour plots showing IDO1 and PD-L1 expression in unstimulated and IFNγ-stimulated condition (concatenation of viable monocytes of 10 unstimulated and stimulated samples). **(C)** Kaplan-Meier estimate of PFS stratified according to low IDO1 upregulation (<457) or high IDO1 upregulation (>457) in CD14^+^ monocytes. *P* value calculated using Log-Rank test.

After incubating PBMCs with IFNγ, a potent IDO1-inducer, IDO1 was upregulated in total monocytes (**Figure 4A**) and in the classical and intermediate monocyte subsets (**Figure 4B**). In contrast, non-classical monocytes did not upregulate IDO1 upon IFNγ stimulation. The magnitude of the IFNγ-induced IDO1 shift varied across patients and a simultaneous upregulation of PD-L1 was observed in both monocyte subsets.

As this shift was clearly heterogeneous across patients, we investigated whether the IFNγ-induced IDO1 upregulation in CD14^+^ monocytes was associated with clinical outcome. Patients’ monocyte response to IFNγ was categorized into low IDO1 upregulation (below median) *versus* high IDO1 upregulation (above median). Strong upregulation of IDO1 in CD14^+^ monocytes was significantly associated with prolonged PFS (Log-Rank test, p=0.008, **Figure 4C**). IDO1 upregulation in CD14^+^ monocytes remained a positive prognostic factor independent of disease stage (**Supplementary Table 1**).

### 3.4 Relation Between Serum, Tissue and Peripheral Blood

In the previous sections, a prognostic role for the serum Kyn/Trp ratio and the basal IDO1 expression and its IFNγ-induced upregulation in circulating CD14^+^ monocytes was observed. In addition, IDO1 expression in the lymph node was associated with altered systemic immune cell frequencies including elevated monocytes. Therefore, we questioned whether there is a relation between the serum Kyn/Trp and IDO1 expression in immune cells of the lymph node and blood compartment.

IDO1 expression in the paracortex and in the sinuses of the lymph node was not associated with the serum Kyn/Trp ratio (resp. p=0.382 and p=0.678, Mann Whitney U test). Similarly, neither serum Kyn/Trp ratio nor IDO1 expression in the lymph node correlated with basal MFI of IDO1 in circulating CD14^+^ monocytes (resp. Spearman’s CC: 0.001, p=0.997; Mann Whitney U test: paracortex: p=0.228, sinus: p=0.112).

As already mentioned, high serum Kyn/Trp as well as high basal IDO1 expression in CD14^+^ monocytes were both associated with worse clinical outcome. When classifying patients into 4 categories depending on their Kyn/Trp ratio (low/high) and basal monocyte IDO1 expression (low/high), none of the 9 patients with a low Kyn/Trp ratio and a low basal IDO1 expression in CD14^+^ monocytes relapsed during follow-up as opposed to 5 out of 11 patients (45.5%) if both parameters were high (Log-Rank test, p=0.140, **Supplementary Figure 4A**).

When patients were categorized according to their serum Kyn/Trp ratio and IFNγ-induced IDO1 upregulation in CD14^+^ monocytes, patients with a low serum Kyn/Trp ratio and a high IDO1 upregulation had the best prognosis as no progressive disease was observed in this subset of patients (0/10) as opposed to 7 out of 13 patients (53.8%) in the subgroup with a high Kyn/Trp ratio and a low IDO1 upregulation (Log-Rank test, p=0.052, **Supplementary Figure 4B**).

**Figure 5:**
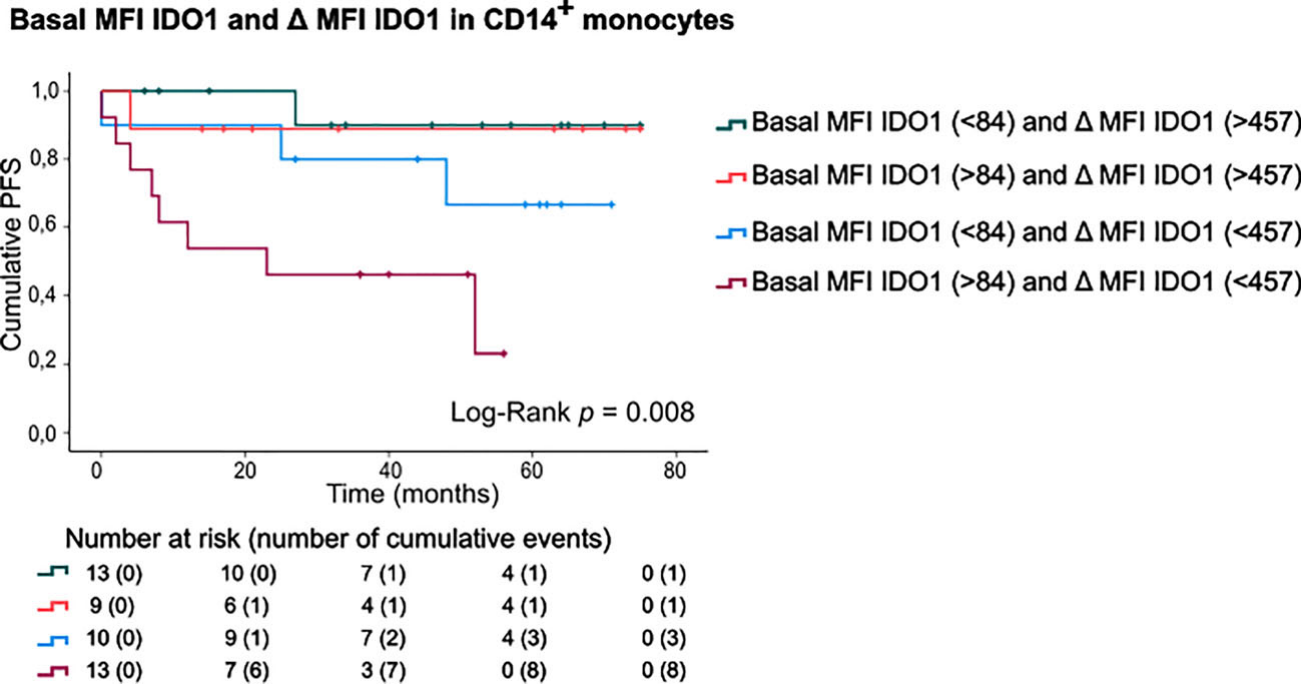
PFS according to basal MFI of IDO1 combined with IFNγ-induced IDO1 upregulation in CD14+ monocytes. Kaplan-Meier estimate of PFS stratified according to basal MFI of IDO1 (low/high, FMO was taken into account) and IFNγ-induced IDO1 upregulation (low/high) in CD14^+^ monocytes. *P* value calculated using Log-Rank test.

Finally, patients were categorized according to basal IDO1 expression and IFNγ-stimulated IDO1 upregulation in CD14^+^ monocytes (**Figure 5**). This sub-stratification rendered significantly different PFS rates (Log-Rank test, p=0.008). Patients with a high basal IDO1 expression combined with low IDO1 upregulation upon IFNγ stimulation had substantially shorter PFS (mean PFS: 29.23 months, CI:16.38-42.08) as opposed to patients with low basal IDO1 expression and a high IDO1 upregulation (mean PFS: 70.20 months, CI:61.28-79.13). Of note, if IFNγ-stimulated IDO1 upregulation was high, only 2 out of 22 patients (9.1%) relapsed during follow-up. If IDO1 upregulation was low, 3 out of 10 patients (30%) relapsed in the subgroup with low basal MFI of IDO1 and 8 out of 13 patients (61.5%) in the subgroup with high basal IDO1 expression in CD14^+^ monocytes.

## 4 Discussion

This study focused on the clinical significance of immune cell IDO1 expression in the lymph node and peripheral blood compartment as well as the significance of Trp metabolites in serum of 65 drug-therapy naïve stage I-III melanoma patients.

Serum Kyn/Trp ratio and its dynamics have been related to worse clinical outcome in various cancer types (22, 29–32). In the current study, we detected an increased Kyn/Trp ratio in stage IV melanoma patients as compared to stage I-III melanoma and healthy controls. PFS rates in stage I-III patients with a high Kyn/Trp ratio was lower than in patients with low Kyn/Trp ratio (55 months *versus* 99 months), but Kyn/Trp was not independent from disease stage in multivariate analysis. An elevated Kyn/Trp ratio has been previously linked to larger tumor size and more advanced disease stages (32–34).

Several factors that may affect the serum Kyn/Trp ratio have been described. In this study, no correlation between the Kyn/Trp ratio and IDO1 expression in immune cells could be demonstrated. Trp can also catabolized to Kyn by two other enzymes: tryptophan 2,3-dioxygenase (TDO) and IDO2. In a study profiling Trp metabolism in 928 cancer cell lines, Kyn secretion in the cancer cell media could be attributed to both IDO1 and TDO expression (23). One third of cell lines simultaneously expressed IDO1 and TDO. Upregulated TDO expression in melanoma is reported in multiple studies (35–37), and is known to be induced by cortisol levels which have been observed elevated in melanoma (38, 39).

Degradation pathways can only use Trp in its free form, which corresponds to 5 to 10% of total Trp, while most of Trp is being bound to albumin (40). Several conditions have been reported to influence the concentration of free Trp, including nutritional and pharmacological factors. Free plasma Trp increases with decreased albumin levels. In addition, nonesterified fatty acids (NEFA) affect Trp availability by displacing it from albumin (41). As a consequence, increasing NEFA levels by adrenaline or phosphodiesterase inhibitors will increase free Trp. The upregulation of the enzymes kynurenine hydroxylase and kynureninase also decrease circulating levels of Kyn, thereby lowering the Kyn/Trp ratio (42). In this study, Kyn/Trp ratio was also significantly higher in males as compared to females, and positively correlated with age. Male sex and increasing age have been correlated with worse outcome in melanoma (43). A recent study demonstrated clear sex- and age-specific differences as a result of differential immunoediting, with female and younger patients exhibiting stronger immune selection in their tumors, resulting in stronger anti-tumor immune responses (44).

Additional factors outside the tumor may affect the Kyn/Trp ratio (45). It is likely that germline immune checkpoint molecule polymorphisms impact the reactivity of the host’ immune system in early stage cancer. Evidence emerges that the strength of anti-tumor immune response is an intrinsic characteristic, in part controlled by the inherited genome. This concept was elaborated by a study demonstrating that germline genetics may determine more than half of the genes expressed in the tumor environment, thereby also influencing immune cell infiltration (46). PD-1/PD-L1 polymorphisms were associated with overall cancer susceptibility (47) and a prognostic impact (48). Although less studied, polymorphisms in *IDO1* have been reported to be associated with overall survival in early stage cancer (49, 50). In a cohort of healthy individuals, a polymorphism in the promoter region of *IDO1* was associated with altered serum Trp concentrations (28).

Recent studies have linked gut microbiome diversity to response to checkpoint blockade, suggesting a cross-talk between the gut microbiome and the immune system (51–53). As Trp is supplied by dietary proteins, it is also metabolized by the gut microbiota, raising the question whether the host gastrointestinal metabolism of Trp impacts the systemic availability of Trp and, consequently, the Kyn/Trp ratio in serum (54). Interestingly, prebiotic and probiotic supplements were demonstrated to modulate Trp metabolism as reflected by decreased serum Kyn and Kyn/Trp levels (55). In a small cohort of HIV infected patients, probiotic intervention reduced IDO mRNA expression in PBMCs (56).

The current study further investigated IDO1 expression by immune cells in the lymph nodes and in the peripheral blood. In the lymph nodes, IDO1 expression was observed in the paracortex and in the sinuses. Consistent with other reports, IDO1 expressing immune cells in the lymph nodes of these patients had the morphology of histiocytes (57, 58). No correlation with PD-L1 expression on immune cells in the lymph node was observed, nor was there a correlation with the serum Kyn/Trp ratio and IDO1 expression in peripheral blood monocytes. IDO1 expression in immune cells of the regional lymph nodes -especially in the sinuses- was associated with higher frequencies of circulating monocytes and lower frequencies of lymphocytes. In addition, IDO1 expression by immune cells in the lymph nodes was associated with a less pro-inflammatory cytokine response after T-cell stimulation with anti-CD3/CD28 beads pointing to lower activation of T-cells in general (CD137, IFNγ and TNFα), reduced cytotoxicity of the CD8^+^ T-cells (granzyme B) and less activity of the T-helper subsets (IL-4, IL-17 and IL-21). These findings suggest a possible impact of IDO1 expression in the lymph node compartment on systemic immunity. No association with PFS was observed.

In the peripheral blood, basal IDO1 expression was the highest in the monocyte compartment. A heterogeneous expression pattern of IDO1 was observed among patients. Although evidence on IDO1 expression in the peripheral blood is scarce, it is well recognized that IDO1 can be induced in response to inflammation (22). Mechanistic studies in mice demonstrated that IDO1 as well as PD-L1 upregulation in the melanoma tumor micro-environment depended on CD8^+^ T-cell infiltration and IFNγ production (59). Accordingly, IDO1 was uniquely detected in CD8^+^ T-cell inflamed regions -also termed immunologically “hot” tumors- in contrast to T-cell non-inflamed “cold” tumors. In our study, basal expression of IDO1 correlated with T-cell activation markers (IFNγ, CD137 and TNFα) and with PD-L1 and HLA-DR expression in CD14^+^ monocytes which are both IFNγ inducible proteins. Basal expression of IDO1 in CD14^+^ monocytes was a negative prognostic marker on PFS, independent from Breslow thickness and disease stage. To our knowledge, the clinical significance of IDO1 expression in monocytes of the peripheral blood of cancer patients has not been reported yet. For PD-L1 expression by monocytes, however, a study in lung cancer patients receiving anti-PD-1 demonstrated that PD-L1 expression was higher on HLA-DR^hi^ monocytes and correlated with low PFS (60).

We next analysed IDO1 upregulation upon *ex vivo* stimulation of patients’ PBMCs with IFNγ. IDO1 was upregulated in monocytes but not in lymphocytes. The IFNγ-induced upregulation of IDO1 was highly variable across patients and occurred in classical as well as in intermediate monocytes. Interestingly, patients in whom monocytes highly upregulated IDO1 had a better outcome than patients with low IDO1 upregulation. When patients were stratified according to basal MFI of IDO1 and IFNγ-induced IDO1 upregulation in CD14^+^ monocytes, the subset of patients with high upregulation had favourable prognosis independent of basal IDO1 expression (2/22 (9%) relapses). If IDO1 upregulation was low, clinical outcome in patients with low basal MFI of IDO1 was worse (3/10 (30%) relapses) and very poor in patients with high basal MFI of IDO1 (8/13 (61.5%) relapses). Considering the fact that basal IDO1 expression in monocytes was remarkably variable across patients, low IFNγ-induced IDO1 upregulation in monocytes with a high basal IDO1 expression might indicate that IDO1 expression was already maximal in these monocytes.

The observation that monocytes upregulate IDO1 upon IFNγ stimulation is supported by several *in vitro* studies in which enhanced IDO1 activity was detected in the monocytic cell line THP-1 upon treatment with IFNγ, as measured by decreased Trp levels and increased concentrations of Kyn and downstream metabolites (e.g. anthranilic acid, neopterin,…) (61–63). Several other studies confirm increased Trp to Kyn degradation in monocytes upon *in vitro* IFNγ stimulation (64–66). In monocyte-derived DCs (moDCs) from healthy controls, treatment with IFNγ induced IDO expression/activity and triggered secretion of proinflammatory cytokines during their activation (67). Curiously, when the IFNγ stimulus was removed by reculturing moDCs in fresh medium, their capacity to produce proinflammatory cytokines was severely reduced but IDO competence was maintained. In the light of our findings, the latter study supports the hypothesis that low IDO1 upregulation upon *ex vivo* IFNγ stimulation may indicate that IDO1 upregulation has already taken place *in vivo* in these patients. This may be associated with reduced proinflammatory signaling.

Monocytes are known to be highly plastic with respect to phenotype and depend on various surrounding signals for differentiation (68). Emerging evidence suggests that the tumor-induced systemic environment influences the phenotype of monocytes before their arrival at the tumor site. This may lead to functional alterations in circulating monocytes, such as the acquisition of immunosuppressive activity (69). Importantly, peripheral blood monocytes constitute the major source of tumor-associated macrophages (TAMs) in the tumor micro-environment (70), a population associated with worse clinical outcome in cancer. In early stage colorectal cancer, monocytes were demonstrated to be responsive -more than any other immune cell population in the circulation- to tumor-derived soluble micro-stimuli (14). Monocyte expression profiles were observed to be modified at early disease onset and remained robust over the course of disease progression. In a cohort of early stage breast cancer, patients who relapsed had dysregulated IFNγ-signalling in monocytes at time of diagnosis (62). These data underline the potential biomarker role of circulating monocytes for progressive disease during follow-up in early stage cancer.

This study reveals some immune features that are associated with clinical outcome in blood samples collected within the first year (median time of blood sampling: 4 months) after diagnosis of local stage and drug-therapy naïve locoregional melanoma. In recent years, patients with resected stage III melanoma have become eligible for adjuvant therapy with immune checkpoint blockade or BRAF-targeted therapy in case of BRAF mutation. Clinical trials exploring these treatments in earlier cancer stages (stage IIB, IIC) are underway. As already mentioned immune failure is one of the hallmarks of cancer development and progression. Further insights into immune dysfunctions in early stage cancer can lead to better patient sub-stratification and more importantly can help to identify possible targets for early intervention.

We acknowledge that the relative small sample size of this study urges its validation in larger cohorts. The retrospective design and the use of cryopreserved blood samples may also represent a limitation. However, this approach allowed us to analyse all samples in the same run (eliminating batch-to-batch variation) and to connect the experimental data with long-term clinical outcome. The necessity of long-term follow-up was evidenced in a large cohort of melanoma patients, in which progression occurred after 5 years or more in 18.2% of progressing patients (71). Biomarkers that allow earlier identification of patients who have higher risk to progress pave the way to personalized management.

## Data Availability Statement

The original contributions presented in the study are included in the article/**Supplementary Material**. Further inquiries can be directed to the corresponding author.

## Ethics Statement

The studies involving human participants were reviewed and approved by Ghent University Hospital. The patients/participants provided their written informed consent to participate in this study.

## Author Contributions

AM, AD, and LB designed the study. NS, MS, PO, and LB provided patient samples and clinical information. AM, AD, and BH conducted experiments. LF and MH evaluated the immunohistochemical staining. AM performed statistical evaluations and created the figures in the manuscript. AD, DA, and LB provided scientific advice. AM and LB wrote the paper with input from AD and the other authors. All authors contributed to the article and approved the submitted version.

## Funding

This work was supported by the Innovation and Clinical Research Foundation of Ghent University Hospital. AM was funded by Kom op tegen Kanker (Stand up to Cancer, grant number 12294), the Flemish cancer society.

## Conflict of Interest

NS reported travel grants from Merck Sharpe & Dohme, Astellas, Bayer and Bristol-Myers Squibb. PO received a research grant from Ferring, Merck, Varian, Bayer, consultancy fees from Ferring, Bayer, Janssen, Sandoz, Sanofi. MH was employed by Dermpat.

The remaining authors declare that the research was conducted in the absence of any commercial or financial relationships that could be construed as a potential conflict of interest.

## Publisher’s Note

All claims expressed in this article are solely those of the authors and do not necessarily represent those of their affiliated organizations, or those of the publisher, the editors and the reviewers. Any product that may be evaluated in this article, or claim that may be made by its manufacturer, is not guaranteed or endorsed by the publisher.

## Supplementary Material

Supplementary Figure 1 Gating strategy of monocyte subsets.

Supplementary Figure 2 Tryptophan and its metabolites according to disease stage. **(A)** Serum concentrations of Trp and Kyn in stage I-IV melanoma and healthy controls. **(B)** Serum concentrations of kynurenic acid (KynA), 5-hydroxyindoleacetic acid (5OHIAA), indole acetic acid (IAA), indole lactic acid (ILA), 5-hydroxytryptophan (5OHTrp) and serotonin (5HT) in stage I-IV melanoma. *P* value calculated using two-sided Mann-Whitney U test. NS, non-significant; *p < 0.05, ***p < 0.001. Reported *p* values are adjusted by Bonferroni correction.

Supplementary Figure 3 Representative immunohistochemical images for PD-L1 expression in immune cells in the lymph nodes. **(A)** PD-L1 was detected in immune cells in the paracortex and **(B)**. in the sinuses.

Supplementary Figure 4 PFS according to serum Kyn/Trp ratio, basal MFI of IDO1 and IFNγ-induced IDO1 upregulation in CD14^+^ monocytes. **(A)** Kaplan-Meier estimate of PFS stratified according to serum Kyn/Trp ratio (low/high) and basal MFI of IDO1 (low/high, FMO was taken into account) in CD14^+^ monocytes. **(B)** Kaplan-Meier estimate of PFS stratified according to serum Kyn/Trp ratio (low/high) and IFNγ-induced IDO1 upregulation (low/high) in CD14^+^ monocytes. *P* value calculated using Log-Rank test.
