# Supplementary figures and images for: Clinical Relevance of Serum Kyn/Trp Ratio and Basal and IFNγ-Upregulated IDO1 Expression in Peripheral Monocytes in Early Stage Melanoma
Source: Front Immunol. 2021 Sep 7;12:736498. doi: 10.3389/fimmu.2021.736498 (PMC8453201; doi:10.3389/fimmu.2021.736498)

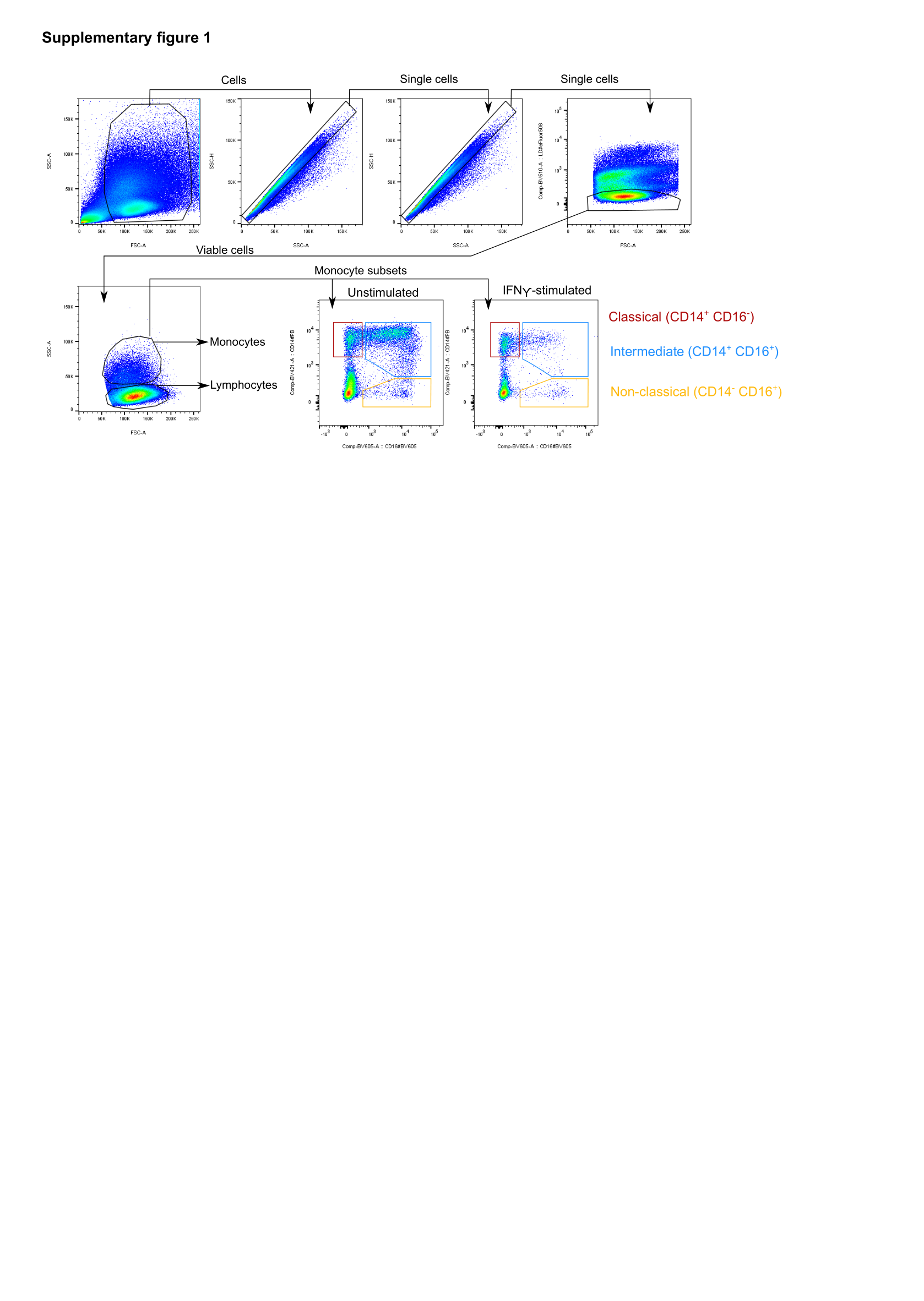

Supplement: Supplementary Figure 1 — Gating strategy of monocyte subsets. [file Image_1.tiff]

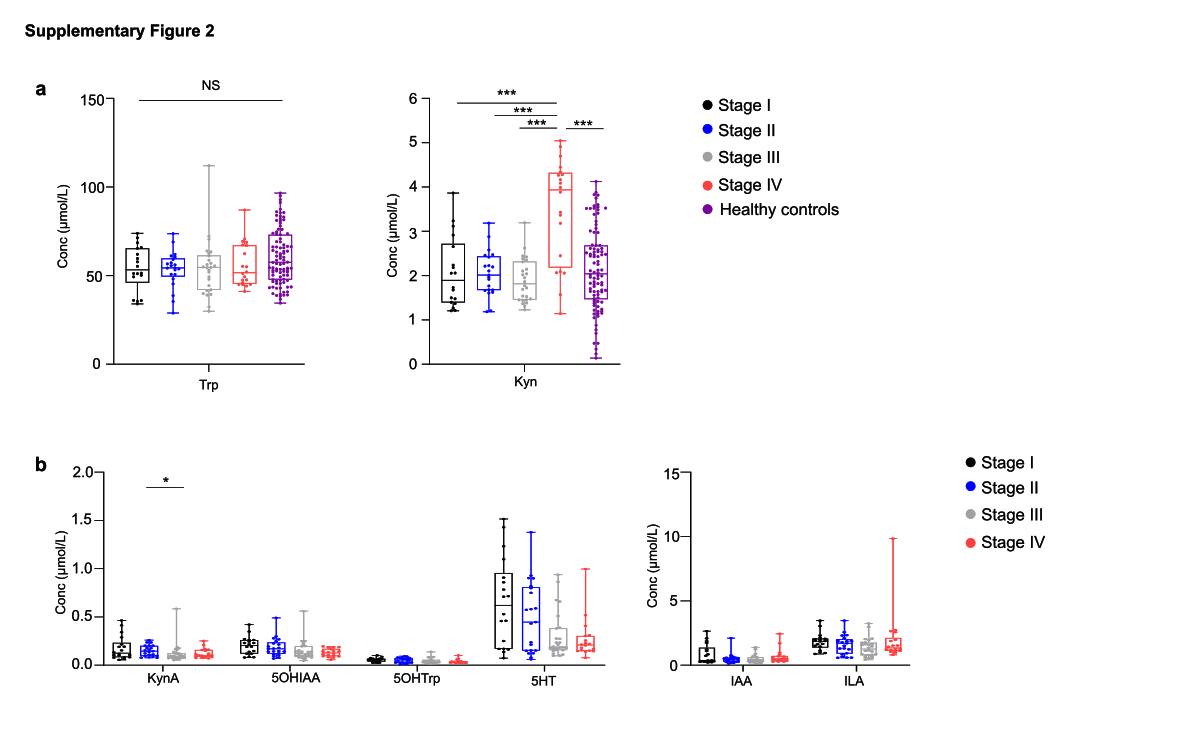

Supplement: Supplementary Figure 2 — Tryptophan and its metabolites according to disease stage. (A) Serum concentrations of Trp and Kyn in stage I-IV melanoma and healthy controls. (B) Serum concentrations of kynurenic acid (KynA), 5-hydroxyindoleacetic acid (5OHIAA), indole acetic acid (IAA), indole lactic acid (ILA), 5-hydroxytryptophan (5OHTrp) and serotonin (5HT) in stage I-IV melanoma. P value calculated using two-sided Mann-Whitney U test. NS, non-significant; *p < 0.05, ***p < 0.001. Reported p values are adjusted by Bonferroni correction. [file Image_2.tiff]

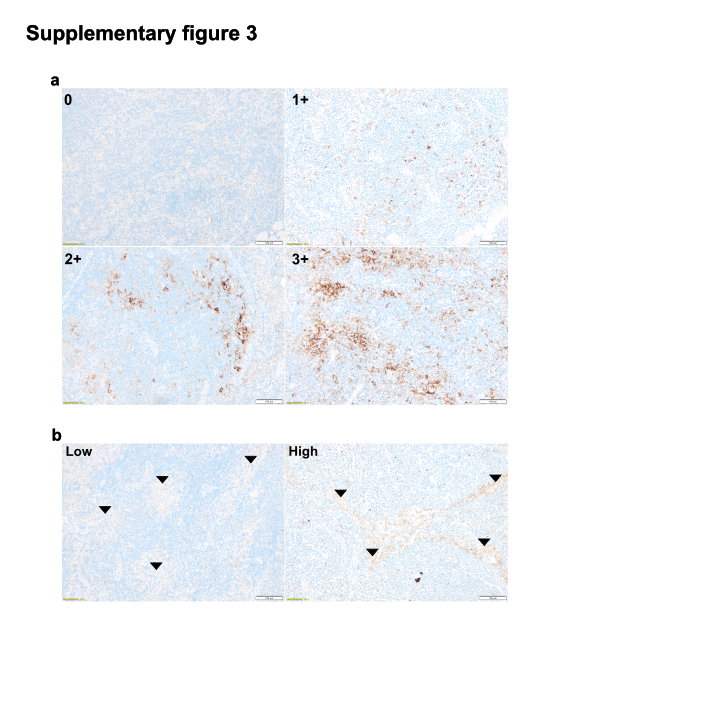

Supplement: Supplementary Figure 3 — Representative immunohistochemical images for PD-L1 expression in immune cells in the lymph nodes. (A) PD-L1 was detected in immune cells in the paracortex and (B). in the sinuses. [file Image_3.tiff]

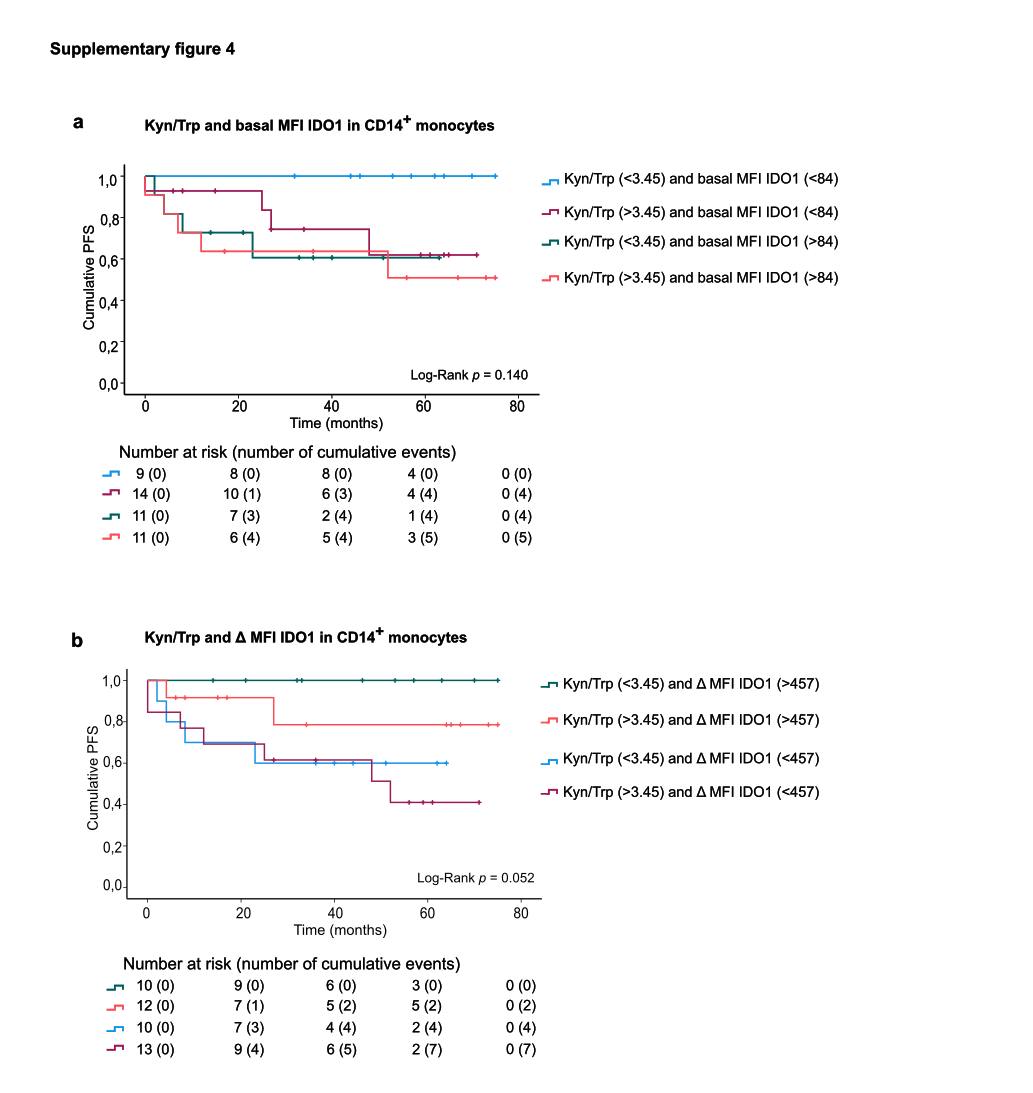

Supplement: Supplementary Figure 4 — PFS according to serum Kyn/Trp ratio, basal MFI of IDO1 and IFNγ-induced IDO1 upregulation in CD14+ monocytes. (A) Kaplan-Meier estimate of PFS stratified according to serum Kyn/Trp ratio (low/high) and basal MFI of IDO1 (low/high, FMO was taken into account) in CD14+ monocytes. (B) Kaplan-Meier estimate of PFS stratified according to serum Kyn/Trp ratio (low/high) and IFNγ-induced IDO1 upregulation (low/high) in CD14+ monocytes. P value calculated using Log-Rank test. [file Image_4.tiff]
