# Supplementary material for: Clinical Relevance of Serum Kyn/Trp Ratio and Basal and IFNγ-Upregulated IDO1 Expression in Peripheral Monocytes in Early Stage Melanoma
Source: Front Immunol. 2021 Sep 7;12:736498. doi: 10.3389/fimmu.2021.736498 (PMC8453201; doi:10.3389/fimmu.2021.736498)
Supplement: Supplementary file 5 [file Table_1.docx]

| **Supplementary Table 1. Multivariate analysis** | |  |  |  |  |  |
| --- | --- | --- | --- | --- | --- | --- |
|  |  |  |  | **95% CI for HR** | |  |
| **Progression free survival** | | **Coefficient** | **HR** | **Lower** | **Upper** | ***p* value** |
| Serum Kyn/Trp | | 0.449 | 1.567 | 1.061 | 2.316 | 0.024 |
| Breslow thickness | | 0.330 | 1.392 | 1.183 | 1.636 | <0.001 |
| Serum Kyn/Trp | | 0.337 | 1.401 | 0.973 | 2.017 | 0.069 |
| Disease stage IA, IB, IIA, IIB, IIIA versus IIC, IIIB, IIIC | | -1.382 | 0.251 | 0.100 | 0.632 | 0.003 |
| Unstim MFI IDO1 in CD14^+^ monocytes (low versus high) | | -2.327 | 0.098 | 0.017 | 0.548 | 0.008 |
| Breslow thickness | | 0.496 | 1.642 | 1.197 | 2.254 | 0.002 |
| Unstim MFI IDO1 in CD14^+^ monocytes (low versus high) | | -1.235 | 0.291 | 0.087 | 0.968 | 0.044 |
| Disease stage IA, IB, IIA, IIB, IIIA versus IIC, IIIB, IIIC | | -1.680 | 0.186 | 0.059 | 0.584 | 0.004 |
| Δ MFI IDO1 in CD14^+^ monocytes (low versus high) |  | 1.386 | 3.998 | 0.845 | 18.912 | 0.081 |
| Breslow thickness | | 0.212 | 1.237 | 0.980 | 1.560 | 0.073 |
| Δ MFI IDO1 in CD14^+^ monocytes (low versus high) | | 1.585 | 4.829 | 1.058 | 22.042 | 0.042 |
| Disease stage IA, IB, IIA, IIB, IIIA versus IIC, IIIB, IIIC | | -1.364 | 0.256 | 0.082 | 0.797 | 0.019 |
